# Stochastic modelling of intersectional pay gaps in universities

**DOI:** 10.1098/rsos.230615

**Published:** 2023-10-11

**Authors:** Tessa Barrett-Walker, Franca Buelow, Lindsey Te Atu O Tu MacDonald, Ann Brower, Alex James

**Affiliations:** ^1^ School of Mathematics and Statistics, University of Canterbury, Christchurch, New Zealand; ^2^ School of Politics and Political Science, University of Canterbury, Christchurch, New Zealand; ^3^ School of Earth Sciences and Environment, University of Canterbury, Christchurch, New Zealand

**Keywords:** ethnicity pay gap, gender pay gap, stochastic model

## Abstract

The gender and ethnicity pay gaps are well publicised for academics. The majority of research relies on observations representing a point in time or uses models to consider a standard academic lifespan. We use a stochastic mathematical model to ask what drives differences in lifetime earnings of university academics and highlight a new question: how best should we quantify a working lifetime? The model observes and accounts for patterns in age when entering and leaving the workforce, and differing salary trajectories during an academic career. It is parameterized with data from a national dataset in Aotearoa New Zealand. We compare the total lifetime earnings of different gender and ethnicity groups with and without accounting for the different lengths of time spent in academia. The lifetime earnings gaps are considerably larger when we account for different hiring and leaving ages. We find that overall, for every ethnicity, women have shorter careers and are more likely to leave academia. All minority ethnic groups—and women—earn considerably less than their male white, European colleagues.

## 1. Introduction

There are three ‘levers’ universities can pull to establish and maintain diversity and equity in the workforce: recruitment, promotion and retention [1]. Diversity includes variations in culture, ethnicity, religion, age, gender and sexual orientation, while equity includes inclusive participation and the removal of barriers to such participation [2]. Many studies have commented on the under-representation of women and ethnic minorities [3,4] in the higher echelons of academia [5–7]. Gender and cultural biases affect all three levers of change [8–10], and present one of several argumentative avenues to explain why and when these levers do have an effect on diversity and equity in academia [11]. Much research focusses on promotion patterns [4,12–14] or promotions and hiring patterns [15].

Fewer studies focus on retention, and its flip-side, attrition [16–19], and within these few, there is little attention given to intersectionality [9]. Studies in United States-based medical faculties have found retention of women and people of colour is considerably lower than for white men [20]. Attrition rates, which are influenced by gender, display a varying degree of magnitude across different ranks, fields and institutions [19]. It has been observed that the attrition rates driven by gender are more pronounced among the tenured faculty, faculty members in non-STEM domains, and those affiliated with lower-prestige institutions [19]. Shaw *et al*. [9] developed a model to simulate ‘predictions’ of the representation they would expect of each federally categorized racial/ethnic group in each stage of academia under the null assumption of no race/ethnicity-based differences in retention and compared the data to actual representations in academia to identify disparities. They suggest that recruiting under-represented scholars is not enough when academia is not equipped to retain them, which highlights the need to identify impacts and extend to which biases affect academic institutions. Clifton *et al*. [21] present a mathematical model that reveals the role that bias and homophily may play in academic careers, and present interventions to achieve gender parity.

Our mathematical model adds to these works and examines hiring and the flip-side of retention, attrition; we also examine intersectionality—asking if there is a different, or compounded, effect for people who fall into two groups outside the majority European-male category (e.g. Māori and Pasifika women). We use a dataset of research performance scores and academic ranks for each researcher employed at every New Zealand university in 2012 and 2018. New Zealand is the only country to score individuals in this way, though other countries score departments or groupings of individuals [7]. We use rank to infer pay using remuneration rates from publicly available academic collective employment agreements. Pay levels are inflation adjusted to 2018 rates. We use this national dataset of individuals' scores dataset to explore the comparative retention rates of European, Asian, Māori, and Pasifika academics in New Zealand universities.

Our preliminary statistical analysis finds, as others have [3,4,15], that men and women of different ethnicities are paid differently and start their academic career at different ages. Further, academic workforce retention is affected by gender, ethnicity and research performance score; these characteristics seem to be intersectional and interact with each other, creating a compounding effect. Such interactions may pose significant challenges when it comes to interpretation and methodology [22] and may give rise to parameter estimates that are deemed unreliable for uncommon combinations of variables owing to the diminishing sample sizes that result from the inclusion of more interactions in the model. While statistical analyses allow preliminary insights into parameters that might affect attrition, the mathematical model we present separates the processes of hiring and attrition and allows us to introduce the effects of retirement. This adds a new dimension, of temporal prediction, to previous point-in-time studies, e.g. [3,4,7,15].

The paper proceeds as follows: after describing the data, we present our statistical analysis, including an overview of hiring patterns and retention. We use this analysis to create a mathematical model connecting hiring, salary and retention patterns. The model allows us to estimate the academic lifetime earnings of an average individual from each group, accounting for gender and ethnic differences in hiring, promotion, and attrition patterns, their compounding effects on lifetime earnings, and any intersectionality effects present. We compare the total lifetime earnings of different gender and ethnicity groups with and without accounting for the different lengths of time spent in academia. The lifetime earnings gaps are considerably larger when we account for different hiring and leaving ages. We conclude with model results, comments on model limitations, and a discussion of our findings.

## 2. Data

We use the Aotearoa New Zealand Performance Based Research Fund (PBRF) datasets from 2012 and 2018. This unique nation-wide dataset included all individuals (*n* = 6253) employed at any of New Zealand's eight universities in either 2012 or 2018. It contains demographic information on gender (male, female), ethnicity (New Zealand European, Māori, Pasifika, Asian or other), age, research performance score and job title. In Aotearoa New Zealand we use different statistical ethnic categories than other countries owing to a different colonization process and, more recently, grounding our constitutional understanding in the signing of the treaty of Waitangi [23]. Māori and Pasifika ethnicities were combined as in some cases the numbers were so small as to violate anonymity.

Research performance score (0–700) is the recommendation of a subject specific panel of experts and claims to be holistic, emphasizing quality over quantity [24]. Scores are converted to grades (0–400: R/C, 400–600: B, 600–700: A) which are reported to individuals and the universities. Academic rank (lecturer; senior lecturer; associate professor; professor) was inferred from job title and converted to salary using 2018 employment agreements. In other words, any salary increases between 2012 and 2018 in the dataset are attributable to promotion (from senior lecturer to associate professor, for example), not to annual pay rises in the collective agreement. We assumed all individuals were on the published salary scales. An unknown number of staff do negotiate individual agreements, hence our assumption probably underestimates the pay for some, probably at the top end. Individuals for whom a rank could not be determined from job title at both time points, such as clinical director or research fellow, were excluded.

Initially the data included 9652 individuals. The data collection did not include a nonbinary gender option, and a small number of individuals had gender not stated at both time points. After removing people for whom rank could not be determined the dataset was reduced to 7711 individuals. Individuals with unstated, i.e. *other*, ethnicity were also excluded reducing the size to 6253 individuals. At this point all individuals had a stated gender either male or female for at least one time point. If ethnicity and gender changed between time points, they were assigned as an individual's most recent declaration, i.e transgender individuals are included at all time points as their last self-reported gender. Including *other* ethnicity as a separate category made little difference to the analysis as this group showed results comparable to the New Zealand European group.

## 3. Statistical analysis

### 3.1. Retention

Individuals who were tenured in 2012 but not present in the dataset in 2018 were assumed to have left the workforce. Their leaving age was taken to be their age at the midpoint of 2015.

### 3.2. Hiring

Individuals who were tenured in 2018 but not in the dataset or of indistinguishable rank in 2012 were assumed to have been hired in the intervening years. Their hiring age was taken to be their age at the midpoint of 2015.

### 3.3. Adjusting for age, score, and gender

We know that research performance score changes with age, increasing more rapidly at the start of a career [7] and, on average, men and women have different scores. This leads us to model the relationship between research score and age and gender using a linear regression model:

$$\mathrm{score}∼{\mathrm{age}}^{2}+\mathrm{gender}.$$

Both variables are significant (*p* < 0.05) when the model is applied to the full dataset of either 2012 or 2018 data.

Following the methodology of [7] we aim to predict the expected lifetime earnings of an individual. We infer salary from rank (available in the PBRF data) and assume that individuals with different research performance scores may take different salary tracks through their lifetime. As with score we allow salary to change more rapidly at the start of an individual's career. We model the relationship of salary with age, gender, and score in a similar fashion to score using

$$\mathrm{salary}∼{\mathrm{age}}^{2}+\mathrm{gender}+\mathrm{score}.$$

All variables are significant (*p* < 0.05) when the model is applied to either the 2012 or 2018 data.

### 3.4. Salary and lifetime earnings

Using the score model above we calculate an individual's expected, i.e. mean average, score given their age and gender. We then use this to calculate their predicted salary. This gives an individuals' salary adjusted for age, gender, and score. Lifetime earnings is the total expected earnings for an individual who starts work at age 30 and retires at age 65 having followed the expected score-salary trajectory throughout their career using the 2018 model output.

### 3.5. Ethnicity

To explore the role of ethnicity we split the dataset by ethnicity group and analyse each subset separately. Where appropriate, we test the validity of this approach by using models on the full dataset, including ethnicity as a predictor variable, and reporting the significance. This approach, of analysing each dataset separately by ethnicity, is akin to using ethnicity as a predictor variable with interactions. Choosing this approach allows us to minimize our assumptions on how ethnicity interacts with other variables.

Table 1 shows a summary of the data, giving mean values of age, research score, and salary for the entire workforce calculated from the raw data in both 2012 and 2018 separately. We also give the expected, i.e. mean average, research score of a 50-year-old individual and the expected salary for a 50-year-old individual with the expected research score. We chose age 50 as an example age close to the average academic. All analysis accounts for the varying age of individuals. Finally, table 1 shows the expected lifetime earnings, expected salary, and expected research score given full employment from age 30 to 65.

**Table 1.** Summary statistics of the data. (Number of individuals, expected age, salary and research score by ethnic group and gender. Score figures in brackets are adjusted for an individual age of 50. Salary figures in brackets are adjusted for an individual age of 50 with the expected research score for that gender/ethnicity. Lifetime earnings is total pay from age 30 to 65 for an individual on the expected score trajectory. Change is the percentage difference between 2012 and 2018. Gap is the relative difference in 2018 between this category and European men.)

		European		Asian		Māori and Pasifika	
	year	F	M	F	M	F	M
*n*	2012	1572	2414	136	306	177	153
	2018	1706	2267	203	388	215	159
	change	+8.5%	−6.1%	+49%	+26%	+21.5%	+3.9%
average age	2012	49.6	51.6	45.5	47.3	47.6	49.7
	2018	51.2	52.9	45.8	46.8	49.4	50.5
	change	+3.2%	+2.5%	+0.7%	−1.1%	+3.8%	+1.6%
average research score (age adjusted at 50)	2012	388 (393)	443 (447)	331 (357)	403 (422)	367 (384)	395 (408)
	2018	411 (420)	465 (473)	369 (395)	416 (438)	425 (436)	433 (444)
	change	+5.9% (+6.9%)	+5.0% (+5.8%)	+11.5% (+10.6%)	+3.2% (+3.8%)	+15.8% (+13.5%)	+9.6% (+8.8%)
	gap 2018	−11.6% (−11.2%)	– (–)	−20.6% (−16.5%)	−10.5% (−7.4%)	−8.6% (−7.8%)	−6.9% (−6.1%)
average salary ($NZ1000) (age adjusted at 50)	2012	117 (119)	131 (131)	108 (114)	118 (122)	107 (112)	117 (121)
	2018	120 (122)	132 (132)	107 (114)	118 (125)	113 (116)	116 (117)
	change	2.6% (2.5%)	0.8% (0.8%)	−0.9% (0%)	0% (2.5%)	5.6% (3.6%)	−0.9% (−3.3%)
	gap 2018	−9.1% (−7.6%)	– (–)	−18.9% (−13.6%)	−10.6% (−5.3%)	−14.4% (−12.1%)	−12.1% (−11.4%)
lifetime earnings, age 30-65 ($NZ million)	2012	4.08	4.50	3.94	4.25	3.81	4.13
	2018	4.14	4.51	3.93	4.30	4.0	4.05
	change	1.5%	0.2%	−0.3%	1.2%	5.0%	−1.9%
	gap	−8.2%	–	−12.9%	−4.7%	−11.3%	−10.2%

The biggest pay increases, after accounting for age and score, from 2012 to 2018 went to Māori and Pasifika women, followed by European women. As 2012 salaries were calculated at 2018 rates, these pay increases are owing to the average individual being employed at a higher rank rather than inflation or similar.

Expected lifetime earnings, assuming a full-time career from age 30 to 65, increased for most groups between 2012 and 2018. Māori and Pasifika women experienced the biggest increase in expected lifetime earnings (5%), though they still had the lowest overall earnings. Overall, the lifetime earnings gap between ethnicities is comparable to the gap between men and women. Asian men, followed by European women had the smallest lifetime earnings gap; and Māori and Pasifika women and Asian women had the largest gap compared to European men.

Between 2012 and 2018, the number of faculty in the dataset, grew by almost 4%. This growth was coincident with an increase in diversity: the number of women increased by 12%, while the number of men fell by 2%. European men saw the largest fall in numbers, whereas the number of Asian and Māori and Pasifika men increased. Similarly, the largest increases of women identified as Asian, and Māori and Pasifika. However, for every ethnicity in 2018, men are slightly older than women, have higher research scores, and earn more. Even after accounting for age, men have higher research scores and earn more than women of the same ethnicity.

After adjusting for age differences, all groups experienced an increase in expected research score at age 50 between 2012 and 2018. Women increased their scores more than men; and Māori and Pasifika saw the largest score increases, 13.5% for women and 8.8% for men, across the different ethnicities. This most likely reflects the increased efforts of the scoring body (Tertiary Education Commission) to recognize Māori and Pasifika research [24].

## 4. Hiring patterns

**Figure 1.**
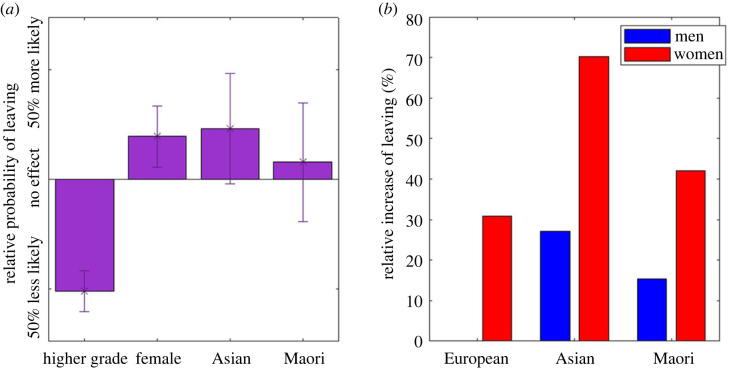
Women, people with lower research performance grades, and non-Europeans are more likely to leave the academic workforce. (*a*) Hazard ratio for each co-variate effect on the likelihood of leaving the academic workforce. Error bars show the 95% confidence interval. (*b*) Hazard ratio for a 50-year-old individual (red, women; blue, men) with a research score typical of their age, ethnicity and gender in comparison to a European male.

We now turn to the data for new hires—those who joined the workforce between 2012 and 2018 (table 2; figure 1). The dataset does not detail which year these individuals were hired so we assume all new hires joined the workforce in 2015. New hires are younger, have lower research scores, and are paid less than their more established colleagues. This implies that most new hires join the workforce at the lower (lecturer) ranks, rather than transferring from overseas at the higher ranks, though without rank when hired (as opposed to rank in 2018 which is used here) we cannot say this conclusively.

**Table 2.** Nationally women join the workforce later than men with a lower research score and lower salary. (Summary statistics for individuals who joined the workforce between 2012 and 2018. Number of individuals, expected age, salary and research score by ethnic group and gender (blue, men; red, women). Significant differences between men and women (two-sided *t*-test) are shown as: (***) *p* < 0.001; (**) 0.001 < *p* ≤ 0.01; (*) 0.01 < *p* ≤ 0.05; (.) 0.05 < *p* < 0.1. Score figures in brackets are adjusted for an individual age of 40. Salary figures in brackets are adjusted for an individual age of 40 (close to the average age of a newly hired academic) with the expected research score for that gender/ethnicity.)

	European		Asian		Māori and Pasifika	
	F	M	F	M	F	M
*N*	614	592	105	162	92	57
mean age when hired	43.2^(*)^	41.8^(*)^	37.5	36.1	43.1	41.1
research score when hired (age adjusted at 40)	348^(***)^ (362)	410^(***)^ (422)	342^(.)^ (365)	371^(.)^ (388)	378 (369)	379 (374)
salary when hired ($NZ1000) (age adjusted at 40)	104^(***)^ (104)	111^(***)^ (112)	96^(**)^ (101)	103^(**)^ (109)	101 (98)	101 (99)

In all ethnicity groups, women join the workforce later than men; but Asian women and men are hired younger than European and Māori and Pasifika. Women, on average, have a lower research score and a lower starting salary than their male counterparts. This difference is much smaller for Māori and Pasifika men and women. After correcting for age, women have similar starting research scores regardless of ethnicity, yet European women have a higher starting salary than Asian or Māori and Pasifika women. Conversely, European men have higher initial scores than Asian or Māori and Pasifika men and higher starting salaries.

In all groups the mean age when hired is surprisingly high. At first this could be read as implying that for many individuals who are hired this is their second, or later, academic job. However, when we compare these ages with available figures for PhD graduation age (a requirement for almost all New Zealand academic jobs) we see this may not be the case. For example, the National Science Foundation records the median PhD graduation age in 2016 [25] as 31.6 years old (slightly higher for women). In fields like education, 40% of PhD graduates were over 40 years old. When we consider that many academics will have at least one postdoctoral position (not included in this study) our findings seem in line with this.

## 6. Attrition

We use Cox's proportional hazard model to estimate the effect of various covariates on an individual's probability of leaving the academic workforce at a particular age (coxphfit, Matlab 2020b) (figure 1). We assume individuals that were present in the 2012 data but absent in 2018 have left the workforce. As the exact leaving date is not included in the data, we assume all individuals left in 2015. Using the full dataset of all ethnicities, we test the effect of gender, ethnicity and research score in 2012 and report the hazard ratio (HR) for the covariates. The model assumes that the different covariates are all independent. This analysis uses one continuous variable (score) and three categorical variables. Although women have lower scores (and Asian women even more so) there are enough individuals in each group with a range of scores that the assumption of independence is not violated. Individuals present in both 2012 and 2018 were included as right censored data. The baseline hazard function is the empirical Kaplan-Meier estimate of the full dataset, other baseline functions were tested and the results were not dependent on the choice.

Figure 1*a* shows the HRs for the covariates. Having a 200 point, i.e. approximately one grade, higher research score was associated with an approximately 50% lower probability of leaving the workforce (HR single point difference: 0.9979, *p* = 10^−27^). Conversely, independent of research performance score, women were 15% more likely to leave (HR: 1.149, *p* = 0.016). After accounting for age, score, and gender neither Asian nor Māori and Pasifika ethnicity was significantly different from European at the 5% level but identifying with Asian ethnicity was significant at the 10% level (*p* = 0.071).

Using the Cox model, effects are assumed to be independent of each other. However, all people have intersectional characteristics and for some, for example an Asian woman, this leads to negative effects being compounded. For example, the average European woman's research score is around 50 points lower than a European man's (table 1); this gives her a compound effect of being female and having a lower research score.

We calculated the cumulative HR for individuals with a typical research performance score in each group in comparison to a European man with an average score (figure 1*b*). Taking an Asian woman as an example, viewed independently, being Asian raises the likelihood of leaving by 18%; and being female raises the likelihood of leaving by 15%. Further, her expected 2012 research score at age 50 that is far lower than that of a European male. In total, this results in an overall chance of leaving which is almost 70% higher in each year than the comparable average (and hence higher scored) European male. All genders and ethnicities are more likely to leave than a European male; the biggest difference is for Asian women followed by Māori and Pasifika women.

## 7. Mathematical model

Our data analysis calculated the lifetime earnings of an individual assuming they were in the academic workforce from age 30 to 65. However, our subsequent analysis on retention has shown that retention and hiring patterns show gender and ethnic differences. Our aim here is to use a mathematical model to connect hiring patterns, salary increases, and retention to calculate the academic lifetime earnings of an average individual from each group; in other words, the total expected income they can expect to receive during their career in the academic workforce. This will account for the different ages at which individuals in these groups are hired and the different times at which they leave and uncover the compounding effect this has on overall earnings during an academic career.

We use a discrete time and individual based, stochastic model where individuals join the academic workforce and then have a constant probability of leaving each year. At some age, *A_R_*, which we expect to coincide approximately with retirement, the probability of leaving each year changes and usually increases. Note that retirement at age 65 is not compulsory in New Zealand and many academics continue to work after this age, though often on a part-time basis (not accounted for here). After this changeover age of 65, the probability of leaving each year is once again constant. This retention model has three key parameters: *p*_early_ and *p*_late_ the probability of leaving in any particular year for the early and late career phases respectively and *A_R_* the age at which behaviour changes. We combine this retention model with a hiring model for the age at which an individual enters the workforce, this has some distribution *f*_enter_. We analyse the model with a step size of one year. The model output is a cumulative distribution function that predicts the probability of an individual having left the academic workforce at a given age.

The model can be run as an individual-based simulation model; but it is also simple enough to be analytically tractable. If an individual joins the workforce the probability of them leaving the workforce after *Y* years, i.e. at age *A*_1_ + *Y* is

$$P(\mathrm{leave}\;\mathrm{after}\;Y\;\mathrm{years})={\left(1-p_{\mathrm{early}}\right)}^{Y}p_{\mathrm{early}},$$

provided they have not reached the changeover age *A_R_*, of *A*_1_ + *Y* < *A_R_*. If they leave after the changeover year the probability of leaving the workforce after *Y* years is

$$P(\mathrm{leave}\;\mathrm{after}\;Y\;\mathrm{years}\;0)={\left(1-p_{\mathrm{early}}\right)}^{A_{R}-A_{1}+1}{\left(1-p_{\mathrm{late}}\right)}^{Y-1-(A_{R}-A_{1})}p_{\mathrm{late}}.$$

Combining these, the probability an individual leaves at age *A* given they entered the workforce at age *A*_1_ is

$$P(\mathrm{leave}\;\mathrm{at}\;\mathrm{age}\;A|\mathrm{enter}\;\mathrm{at}\;\mathrm{age}\;A_{1})=\{\begin{array}{cc} {\left(1-p_{\mathrm{early}}\right)}^{A-A_{1}}p_{\mathrm{early}}, & A<A_{R}\\ {\left(1-p_{\mathrm{early}}\right)}^{A_{R}-A_{1}+1}{\left(1-p_{\mathrm{late}}\right)}^{A-A_{R}-1}p_{\mathrm{late}}, & A\geq A_{R} \end{array}.$$

Incorporating the probability of entering the workforce at age *A*_1_ the probability of leaving the workforce at age *A* is

$$P(\mathrm{leaving}\;\mathrm{at}\;\mathrm{age}\;A)=\;\sum_{A_{1}=0}^{A-1}P(\mathrm{enter}\;\mathrm{at}\;\mathrm{age}\;A_{1})\times P(\mathrm{leave}\;\mathrm{at}\;\mathrm{age}\;A|\mathrm{enter}\;\mathrm{at}\;\mathrm{age}\;A_{1})\;.$$

For completeness this is summed over age from birth but the contribution of terms with *A*_1_ < 25 is very small. We fit the model to data by minimizing the root mean square error (RMSE) between the model and data cumulative distribution functions for the probability of having left the workforce at age *A*. For the data this is the empirical Kaplan-Meier estimate (as described in the hazard analysis above). We fitted the model to each gender/ethnicity grouping separately. The probability of entering the workforce at age *A*_1_ is found from a Gamma distribution fitted to the arrival ages for that gender/ethnicity group separately. Model fitting is done over the continuous variables *p*_early_ and *p*_late_ (using Matlab 2020b, lsqcurvefit) and minimizing the RMSE over the discrete variable *A_R_*. Alternative fitting approaches could focus on minimizing the KS-statistic, but this has a tendency to focus on optimizing a single part of the distribution, usually the tail so was not used here. Confidence intervals are found using bootstrapping. We ran 100 bootstrap simulations, each simulation created a new dataset, the same size as the original by sampling individuals from the original dataset with replacement. Confidence intervals are the 95% interval from the output of these simulations.

We define a null model with only a single probability of leaving each year, i.e. *p*_early_ = *p*_late_. This is equivalent to using a standard Poisson process resulting in an exponential distribution of leaving times. We use this as a reference for a modified goodness of fit definition based on the standard Pearson's R-squared:

$$r_{\mathrm{mod}}^{2}=1-\frac{\sum_{i}{\left(y_{\mathrm{dat}}-y_{\mathrm{model}}\right)}^{2}}{\sum_{i}{\left(y_{\mathrm{dat}}-y_{\mathrm{null}}\right)}^{2}}.$$

Like the usual coefficient of determination, $r_{\mathrm{mod}}^{2}=0$ if the model is only as good as the null model and 1 if the model is a perfect fit. Negative values indicate a fit worse than the null model. As our stochastic model has three parameters and includes the null model as a subset, we expect it to always be a better fit than the null model.

Finally, our model can be combined with the statistical earnings model presented earlier. Previously we calculated lifetime earnings assuming an individual entered the academic workforce at age 30 and left at 65. Now we include an individual's expected starting age and their predicted leaving age. This gives the expected academic lifetime earnings which is the total salary averaged over all possible starting ages and leaving ages:

$$\mathrm{academic}\;\mathrm{lifetime}\;\mathrm{earnings}=\sum_{A_{1}=20}^{65}\sum_{A_{2}=21}^{80}P(\mathrm{hired}\;\mathrm{at}\;\mathrm{age}\;A_{1})\times P(\mathrm{leave}\;\mathrm{at}\;\mathrm{age}\;A_{2})\times \mathrm{salary}(A_{1},A_{2}).\;$$

Where salary(*A*_1_, *A*_2_) is the expected lifetime salary of an individual entering the academic workforce at age *A*_1_ and leaving at age *A*_2_. We assume individuals are only hired between the ages of 20 and 65, and all individuals leave at age 80.

## 8. Model results

**Figure 2.**
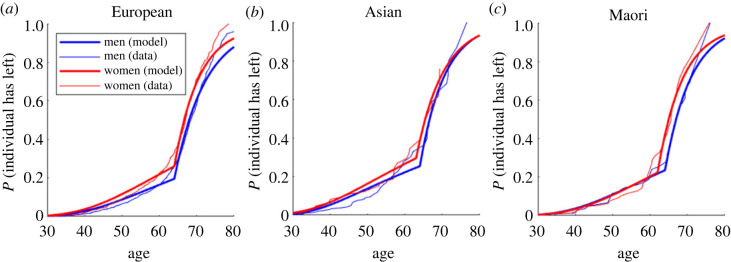
Accounting for differences in joining the workforce as well as leaving provides a good fit for each gender/ethnicity group in both datasets. Model output on the probability an individual has left the workforce by a given age (dark lines) and corresponding data (light lines) for each gender/ethnicity grouping.

Figure 2 shows the model (dark lines) fitted to the data (light lines) split by ethnicity and gender. The model is a good fit to the data although it predicts a slightly more abrupt change of behaviour at the changeover age (see model limitations). For every ethnicity and at almost every age, women (red lines) are more likely to leave the academic workforce than men (blue lines). Table 3 shows the best fit model parameters and confidence intervals.

**Table 3.** Best fit model parameters and key model output. (Figures in brackets are 95% confidence intervals.)

	European		Asian		Māori and Pasifika	
	F	M	F	M	F	M
*p*_early_ (%)	1.44% (1.2, 1.62)	0.970% (0.89, 1.1)	1.37% (0.77, 1.9)	1.04% (0.75, 1.21)	1.32% (0.69, 1.83)	1.17% (0.68, 1.83)
*p*_late_ (%)	14.3% (11.1, 16.5)	11.4% (10.9, 13.6)	13.0% (6.4, 33.4)	14.0% (9.0, 21.2)	14.3% (10.5, 31.5)	13.5% (10.4, 20.2)
changeover age *A_R_* (years)	64 (63, 65)	64 (64, 65)	63 (60, 65)	64 (61, 65)	62 (60, 65)	64 (63, 65)
$r_{\mathrm{mod}}^{2}$	0.943	0.954	0.891	0.910	0.922	0.944
academic lifetime earnings	$NZ 2.7 m (2.6, 2.8)	$NZ 3.3 m (3.2, 3.4)	$NZ 2.9 m (2.5, 3.2)	$NZ 3.5 m (3.2, 3.7)	$NZ 2.5 m (2.1, 2.6)	$NZ 2.9 m (2.7, 3.3)

Before the changeover at retirement, women are more likely to leave than men in every ethnicity group. For the largest group, Europeans, this is significant at the 95% level based on the bootstrapped 95% confidence intervals.

**Figure 3.**
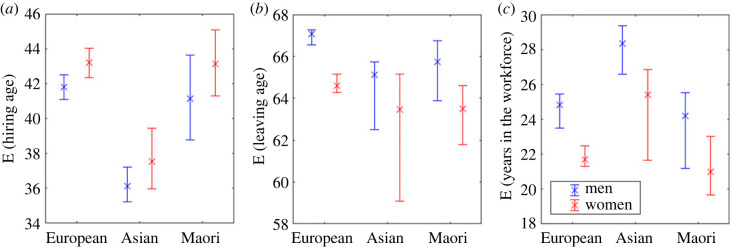
In all ethnicities men leave later than women and overall stay longer in the workforce. (*a*) Expected (mean) hiring age (from data). (*b*) Model predicted mean leaving age and (*c*) model predicted mean years in the workforce. Error bars show 95% confidence interval.

Using the model, we can calculate the mean leaving age for each group and the expected number of years spent in the workforce, assuming arrival age and leaving age are independent (figure 3). Women have shorter careers than men in every ethnicity group. Asian people have the longest careers, mostly owing to their early starting age. Māori and Pasifika and European women have the shortest careers. The academic lifetime earnings, which now account for career span rather than the standard 30-65 years previously assumed, have all decreased from the original estimates, reflecting the shorter working lifetimes predicted by the model.

**Figure 4.**
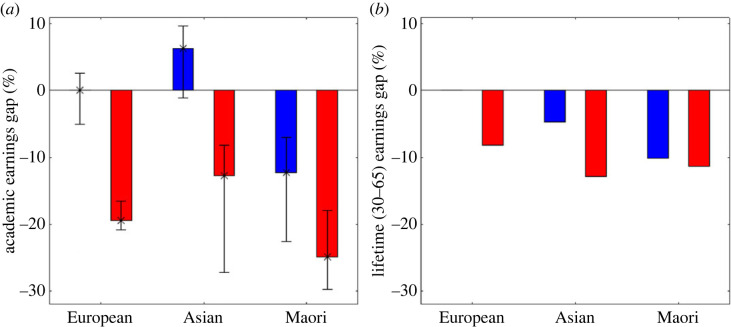
After accounting for time spent in academia the difference in lifetime earnings between different genders and ethnicities are significantly bigger. (*a*) Lifetime earnings relative to a European male accounting for time spent in the academic workforce using the proposed model including hiring age and attrition. (*b*) Lifetime earnings relative to a European male assuming all individuals have the same career span career from age 30 to 65.

Finally, we compare the lifetime earnings of each group, with and without accounting for time spent in academia. Initially we assume all individual's start their career at age 30 and leave at age 65. An individual has the expected research score for their age, gender, and ethnicity and this predicts their expected salary at each year. The total lifetime salary of each gender/ethnicity group is compared to the expected lifetime salary of a European man. We then do the same calculation but using the hiring and leaving distributions predicted by the model. Without accounting for time spent in the academic workforce, i.e. everyone starts at age 30 and leaves at age 65 (figure 4*b*; cf. table 1), most groups earn between 5% and 12% less than their male European colleagues. However, when we account for time spent in academia (figure 4*a*) these differences increase significantly for most groups. Māori and Pasifika women earn almost 25% less than their male European colleagues during the time that they are working in academia, and European women almost 20% less. Conversely, Asian men now earn more, because they are hired earlier and have a longer academic working lifetime.

## 9. Model limitations

A limitation of the model is that we assume joining and leaving the workforce are independent of each other, that joining early does not correlate with leaving either early or late. Collecting this type of data would require long term historical studies to watch individuals over their entire career. Additionally, we would still need to assume, as our model does, that individuals joining now will follow the same patterns as those who joined many years ago.

An alternative modelling approach would be to use a Weibull model or similar statistical function for retention. The downside of this method is that these models may fail to account for the change in behaviour seen around retirement age. For example, an individual hired at age 55 will probably have a marked increase in leaving probability around 10 years after hiring, whereas an individual hired at age 35 will see this sudden increase after 30 years. The model presented here accounts for absolute age as well as number of years since hiring. Most ‘off-the-shelf’ statistical models are unable to do this and would require considerable modification to capture this behaviour.

Given the limited data available we have assumed that individuals were hired or left at the middle of the time period in 2015. There was no mass exodus or hiring of academics from any New Zealand university in the 6 years of the study, so we feel the assumption that hiring and leaving were constant over this period is well founded. Comparing tables 1 and 2 show that, using European females as an example, 600 individuals were hired between 2012 and 2018 and approximately 400 left. So, this approximation has been applied to a large number of people. An alternative approach would be to assign individuals a leaving/hiring date from a uniform distribution over the 6 years. The mathematics of random processes, Poisson processes in particular, predict that this would increase the variance of the results but is very unlikely to affect the mean [26,27]. An increase in the variance of the model outputs would also result in an increase in the variance of the parameter estimates, i.e. the confidence intervals presented in table 3 and figure 3 would be larger.

Most model improvements would increase the number of parameters in the model, hence were not included owing to the limited amount of data for some groups. One obvious model improvement would be to allow the age at which behaviour changes to vary across individuals. This would require an additional parameter for the variance of the changeover age. Separately, the model as it stands predicts a higher number of much older individuals remaining than is seen in the data, showing that at this very late career stage the probability of leaving each year most likely increases in time rather than the constant leaving probability used. This could be accounted for with a Weibull distribution to allow the probability of leaving to increase with time after the changeover age. Again, this would add at least one additional parameter to the model and for some groups there is only a minimal amount of data in this region.

## 10. Discussion

We have presented a model for earnings during an academic career that accounts for variation in starting and leaving ages for individuals of different gender and ethnicity. The model uses national data on all academic staff from Aotearoa New Zealand from 2012 to 2018. It accounts for different salary trajectories based on both age and performance. We consider two definitions of lifetime earnings: total earning assuming a career from age 30 to 65; and total earnings accounting for variation in hiring and leaving age. When we assume a standardized lifespan of 35 years (30–65) all groups have a pay gap compared to European men of between 5% and 12% and women's pay gaps are bigger than men's. When we adjust for variation in hiring and leaving ages the lifetime pay gap for European, and Māori and Pasifika women compared to European men becomes significantly bigger, rising to approximately 20% and 25% respectively.

A much bigger pay gap after account for academic working life span is not necessarily a cause for concern. If academic jobs were poorly paid and leaving early was probably owing to moving on to more lucrative employment, or starting an academic career was associated with a pay cut for most people, then a short academic career could be seen a good thing in terms of entire career earnings. While this will be the case for some it is unlikely to be the majority. A starting academic salary of $NZ80,000 is in the top 13% of earners and a mid-range position in the professoriate (∼$NZ140 000) is in the top 4%. For women in general and Māori and Pasifika men and women these salaries would put them in a higher percentile [28]. One limitation of this comparison is that we do not have access to data on earning of the individuals we analyse here before and after they enter the dataset. An additional consideration is that we have shown a lower research performance score is associated with a higher probability of leaving. At each stage of their career, female faculty members exhibit a higher tendency to resign from their positions in comparison to their male counterparts. This phenomenon is particularly pronounced in institutions that are considered as lower-prestige, non-STEM fields as well as among women who have attained tenure [19]. Moving to a more lucrative academic job outside New Zealand would probably be an opportunity for those with higher research performance scores.

The under representation of staff from a wide range of backgrounds has an effect on students' perception on the interplay of diversity, equity and inclusion and career pathways and options [9,29]. While racial/ethnic and gender inequalities have been discussed broadly and reported widely in the literature, investigations on the way they interact are more recent [30,31]. We find a compounding effect when applying an intersectionality approach to our analysis of lifetime earnings: Overall, we see earlier exits, lower scores, lower earnings, and lower promotion prospects. Based on our data, those seem to be inter-related. This results in a lack of representation of and loss of contributions from women from under-represented minorities, who experience the interplay of sexism and racism [32]. Literature shows that reasons for such dynamics can be based on broader sociological factors such as: difficulty or no access to informal, powerful, and male-dominated networks [33] biases and stereotypes affecting performance evaluation [34], promotion decisions and thus retention and attrition [20]. Other factors that have an effect on career paths and diversity of academic staff include dissatisfaction with salary [7], lack of job satisfaction i.e. owing to lack of diversity management [35], disruptive student behaviour and evaluations [8], excessive workload [34], low turnover and lack of administrative support [36], family involvement and role conflicts [37], recruitment channels, marketing and communication [38] and age and experience in the field [39]. However, our dataset does not include the reason for leaving, which in some cases could be for higher paid employment overseas so we cannot reach direct conclusions.

Our results show that women are more likely to leave academia than men and have shorter careers, regardless of their ethnicity. Other research shows this attrition starts after the completion of the PhD and may increase as women progress on the career ladder [7,15,40]. Women, especially tenured women, are more likely to leave or consider leaving owing to poor workplace climate rather than concerns about work-life balance [19]. Attrition has led to the popular descriptions of a glass ceiling and leaky pipeline [41,42] a predominantly male academic work environment, where quality and potential are more often doubted in female candidates than in male candidates [43,44]. The comparatively early exit and/or late entry might be owing to dissatisfaction with pay and promotion opportunities [45] family obligations, which fall disproportionately on women [46], and motherhood [47]. This is further exacerbated by the isolating effects of being a minority, resulting in lack of mentoring and networks and discrimination [45].

Our model shows that on average, women start their tenured academic career later than men with a lower research performance score; even after accounting for the different ages, women still start with a lower research performance score. This suggests that gendered age differences do not account for differences in career progression or performance score, complementing previous work [7,15]. Structural reasons as well as the impact of biases might account for the differences in performance scores and have been explored in relation to a gendered productivity gap [48], or productivity puzzle [49]. Women expect men to perform better—and men agree [41]. They deal with a mosaic of inequity in financial compensation, grant funding, publications, authorship, citations, and speaking roles [50], all of which seem to be more pronounced when adding race/ethnicity characteristics [12,32].

On top of having the shortest careers, we find that Māori and Pasifika and European women earn 20% less than the average European male colleague when comparing lifetime earnings. Similar results on differences in salaries have been found in the United Kingdom and the United States [51]. There is ample evidence of a persistent gender pay-gap [52] and lack of women in permanent positions [19]. Some say women earn less because they specialise less [53], or shoulder most of parenthood/care-work [48], and are less visible owing to lower publication and promotion rates [52,53]. Although more recent work suggests that overall parents publish more than non-parents [48], it still shows women disadvantaged overall.

The fact that women, especially non-Europeans, are likely to have lower research scores than men, and leave academia earlier and with lower lifetime earnings, results in highly gender-biased leaving rates, particularly for Asian and Māori and Pasifika women. Our analysis once again shows that contrary to the ‘myth of meritocracy’ and the gender neutrality of universities, they are, like other organizations, gendered organizations [43,54]. That is, universities are organizations in which gender permeates all structures, processes and relationships as a constitutive element [55], and in which gender stereotypes, norms, institutional resistance to intersectional equity both produce and reproduce inequality [32]. Seeing women academics from under-represented minorities plateau or leave while European men advance their careers creates a strongly standardizing effect around male-dominated environments, as discussed elsewhere [32,56]. Our findings add to the canon of research that, together, underscores the need to address intersectional disadvantages and to develop effective policies to overcome narrow, gendered hiring, promotion and attrition practices (e.g. [12,32,56,57]).

## Data Availability

The data used in this study are owned by a third-party organization (Tertiary Education Commission (TEC), New Zealand). The authors were granted access privileges to the data, under strict nondisclosure agreements, by the TEC for this research project only, under New Zealand's Official Information Act 1992. The Official Information Act 1992 facilitates New Zealanders' access to government records, through a formal information request. All New Zealand citizens and residents may make such requests, under the following guidelines: https://www.dia.govt.nz/Official-Information-Actrequests. This dataset pertains to thousands of people's employment; hence are strictly private and highly sensitive. Owing to ethical and privacy restrictions, a de-identified dataset cannot be made publicly available. Interested researchers are invited to contact the corresponding authors to discuss access to data. Electronic supplementary material is available online [58].

## References

[1] James A, Brower A. 2022 Levers of change: using mathematical models to compare gender equity interventions in universities. R. Soc. Open Sci. **9**, 220785. (10.1098/rsos.220785) 36133151 PMC9449479

[2] Berry J. 2016 Diversity and equity. Cross Cult. Strategic Manage. **23**, 413-430. (10.1108/CCSM-03-2016-0085)

[3] Mcallister T, Kidman J, Rowley O, Theodore R. 2019 Why isn't my professor Mäori. Mai J. **8**, 235-249.

[4] Naepi S. 2019 Why isn't my professor Pasifika. Mai J. **8**, 219-234.

[5] Cech EA. 2022 The intersectional privilege of white able-bodied heterosexual men in STEM. Science Advances **8**, eabo1558. (10.1126/sciadv.abo1558) 35704581 PMC9200289

[6] Begeny CT, Ryan MK, Moss-Racusin CA, Ravetz G. 2020 In some professions, women have become well represented, yet gender bias persists—perpetuated by those who think it is not happening. Sci. Adv. **6**, eaba7814. (10.1126/sciadv.aba7814) 32637616 PMC7319752

[7] Brower A, James A. 2020 Research performance and age explain less than half of the gender pay gap in New Zealand universities. PLoS ONE **15**, e0226392. (10.1371/journal.pone.0226392) 31967992 PMC6975525

[8] Fan Y, Shepherd LJ, Slavich E, Waters D, Stone M, Abel R, Johnston EL. 2019 Gender and cultural bias in student evaluations: why representation matters. PLoS ONE **14**, e0209749. (10.1371/journal.pone.0209749) 30759093 PMC6373838

[9] Shaw AK, Accolla C, Chacón JM, Mueller TL, Vaugeois M, Yang Y, Sekar N, Stanton DE. 2021 Differential retention contributes to racial/ethnic disparity in US academia. PLoS ONE **16**, e0259710. (10.1371/journal.pone.0259710) 34851964 PMC8635368

[10] Boring A. 2017 Gender biases in student evaluations of teaching. J. Public Econ. **145**, 27-41. (10.1016/j.jpubeco.2016.11.006)

[11] Nelson LK, Zippel K. 2021 From theory to practice and back: How the concept of implicit bias was implemented in academe, and what this means for gender theories of organizational change. Gender Soc. **35**, 330-357. (10.1177/08912432211000335)

[12] Fox Tree JE, Vaid J. 2022 Why so few, still? Challenges to attracting, advancing, and keeping women faculty of color in academia. Front. Sociol. **6**, 238. (10.3389/fsoc.2021.792198) PMC8804352 35118155

[13] Dutt K, Pfaff DL, Bernstein AF, Dillard JS, Block CJ. 2016 Gender differences in recommendation letters for postdoctoral fellowships in geoscience. Nat. Geosci. **9**, 805-808. (10.1038/ngeo2819)

[14] Eaton AA, Saunders JF, Jacobson RK, West K. 2020 How gender and race stereotypes impact the advancement of scholars in STEM: professors' biased evaluations of physics and biology post-doctoral candidates. Sex Roles **82**, 127-141. (10.1007/s11199-019-01052-w)

[15] Walker L, Sin I, Macinnis-Ng C, Hannah K, Mcallister T. 2020 Where to from here? Women remain absent from senior academic positions at Aotearoa New Zealand's universities. Educ. Sci. **10**, 152. (10.3390/educsci10060152)

[16] , Lee M, Coutts R, Fielden J, Hutchinson M, Lakeman R, Mathisen B, Nasrawi D, Phillips N, 2022 Occupational stress in University academics in Australia and New Zealand. J. Higher Educ. Pol. Manage. **44**, 57-71. (10.1080/1360080X.2021.1934246)

[17] Benseman J, Coxon E, Anderson H, Anae M. 2006 Retaining non-traditional students: lessons learnt from Pasifika students in New Zealand. High. Educ. Res. Dev. **25**, 147-162. (10.1080/07294360600610388)

[18] Wapman KH, Zhang S, Clauset A, Larremore DB. 2022 Quantifying hierarchy and dynamics in US faculty hiring and retention. Nature **610**, 120-127. (10.1038/s41586-022-05222-x) 36131023 PMC9534765

[19] Spoon K, Laberge N, Wapman KH, Zhang S, Morgan A, Galesic M, Fosdick B, Larremore D, Clauset A. 2023 Gender and retention patterns among US faculty. 10.1126/sciadv.adi2205 PMC10588949 37862417

[20] Cropsey KL et al. 2008 Why do faculty leave? Reasons for attrition of women and minority faculty from a medical school: four-year results. J. Womens Health (Larchmt) **17**, 1111-1118. (10.1089/jwh.2007.0582) 18657042

[21] Clifton SM, Hill K, Karamchandani AJ, Autry EA, Mcmahon P, Sun G. 2019 Mathematical model of gender bias and homophily in professional hierarchies. Interdiscip. J. Nonlinear Sci. **29**, 023135. (10.1063/1.5066450) 30823713

[22] Bell A, Holman D, Jones K. 2019 Using shrinkage in multilevel models to understand intersectionality. Methodology **15**, 88-96. (10.1027/1614-2241/a000167)

[23] Statistics New Zealand. 2005 Statistical standard for ethnicity 2005. Wellington, New Zealand: Statistics New Zealand.

[24] Tertiary Education Commission. 2016 Performance based research fund (PBRF) user manual, version 4: November 2016.

[25] National Science Foundation National Center for Science and Engineering Statistics. 2018 Doctorate recipients from U.S. Universities: 2016. Alexandria, VA: National Science Foundation.

[26] Lloyd-Smith JO, Schreiber SJ, Kopp PE, Getz WM. 2005 Superspreading and the effect of individual variation on disease emergence. Nature **438**, 355-359. (10.1038/nature04153) 16292310 PMC7094981

[27] James A, Pitchford JW, Plank MJ. 2007 An event-based model of superspreading in epidemics. Proc. R. Soc. B **274**, 741-747. (10.1098/rspb.2006.0219) PMC2197209 17255000

[28] Cochrane WR, Pacheco G, Cochrane B, Pacheco G. 2022 Empirical analysis of Pacific, Māori and ethnic pay gaps in New Zealand. Aukland, New Zealand: New Zealand Work Research Institute.

[29] Nelson Laird TF. 2005 College students' experiences with diversity and their effects on academic self-confidence, social agency, and disposition toward critical thinking. Res. High. Educ. **46**, 365-387. (10.1007/s11162-005-2966-1)

[30] Crenshaw K. 1989 Demarginalizing the intersection of race and sex: a black feminist critique of antidiscrimination doctrine, feminist theory and antiracist politics. u Chi Legal f 139, **1989**, art. 8.

[31] Becares L, Priest N. 2015 Understanding the influence of race/ethnicity, gender, and class on inequalities in academic and non-academic outcomes among eighth-grade students: Findings from an intersectionality approach. PLoS ONE **10**, e0141363. 26505623 10.1371/journal.pone.0141363 PMC4624767

[32] Chellappa SL. 2023 Intersectional inequities in academia. Lancet **401**, 1076. (10.1016/S0140-6736(23)00229-5) 37003692

[33] Bozeman B, Corley E. 2004 Scientists’ collaboration strategies: implications for scientific and technical human capital. Evol. Hum. Behav. **33**, 599-616. (10.1016/j.respol.2004.01.008)

[34] Manchester C, Barbezat D. 2013 The effect of time use in explaining male–female productivity differences among economists. Ind. Relat.: J. Econ. Soc. **52**, 53-77. (10.1111/irel.12011)

[35] Pitts D. 2009 Diversity management, job satisfaction, and performance: evidence from US federal agencies. Public Adm. Rev. **69**, 328-338. (10.1111/j.1540-6210.2008.01977.x)

[36] O'Brien KR, Scheffer M, Van Nes EH, Van Der Lee R. 2015 How to break the cycle of low workforce diversity: a model for change. PLoS ONE **10**, e0133208. 26217957 10.1371/journal.pone.0133208 PMC4517751

[37] Viglione G. 2020 Are women publishing less during the pandemic? Here's what the data say. Nature **581**, 365-366. (10.1038/d41586-020-01294-9) 32433639

[38] Tipper J. 2004 How to increase diversity through your recruitment practices. Ind. Commer. Train. **36**, 158-161. (10.1108/00197850410542392)

[39] Pritchard K, Whiting R. 2015 Generational diversity at work: new research perspectives. Person. Rev. **44**, 176-179. (10.1108/PR-10-2014-0244)

[40] Ledin A, Bornmann L, Gannon F, Wallon G. 2007 A persistent problem: traditional gender roles hold back female scientists. EMBO Rep. **8**, 982-987. (10.1038/sj.embor.7401109) 17972895 PMC2247380

[41] Ollrogge K, Roswag M, Hannover B. 2022 What makes the pipeline leak? Women's gender-based rejection sensitivity and men's hostile sexism as predictors of expectations of success for their own and the respective other gender group. Front. Psychol. **13**, 800120. (10.3389/fpsyg.2022.800120) 36267067 PMC9577486

[42] Van Veelen R, Derks B. 2022 Equal representation does not mean equal opportunity: women academics perceive a thicker glass ceiling in social and behavioral fields than in the natural sciences and economics. Front. Psychol. **13**, 790211. (10.3389/fpsyg.2022.790211) 35369222 PMC8966382

[43] Van Den Brink M, Benschop Y. 2014 Gender in academic networking: the role of gatekeepers in professorial recruitment. J. Manag. Stud. **51**, 460-492. (10.1111/joms.12060)

[44] Madera JM, Hebl MR, Dial H, Martin R, Valian V. 2019 Raising doubt in letters of recommendation for academia: gender differences and their impact. J. Business Psychol. **34**, 287-303. (10.1007/s10869-018-9541-1)

[45] Hunt J. 2016 Why do women leave science and engineering? ILR Rev. **69**, 199-226. (10.1177/0019793915594597)

[46] Ferrant G, Pesando LM, Nowacka K. 2014 Unpaid care work: the missing link in the analysis of gender gaps in labour outcomes. Boulogne Billancourt: OECD Development Center.

[47] O'Brien KR, Hapgood KP. 2012 The academic jungle: ecosystem modelling reveals why women are driven out of research. Oikos **121**, 999-1004. (10.1111/j.1600-0706.2012.20601.x)

[48] Morgan AC, Way SF, Hoefer MJ, Larremore DB, Galesic M, Clauset A. 2021 The unequal impact of parenthood in academia. Sci. Adv. **7**, eabd1996. (10.1126/sciadv.abd1996) 33627417 PMC7904257

[49] Larivière V, Ni C, Gingras Y, Cronin B, Sugimoto CR. 2013 Bibliometrics: global gender disparities in science. Nature **504**, 211-213. (10.1038/504211a) 24350369

[50] Johnson C, Smith JL, Van Laar C. 2022 Women in academia: challenges and solutions to representation in the social sciences.

[51] Barrett L, Barrett P. 2011 Women and academic workloads: Career slow lane or cul-de-sac? High. Educ. **61**, 141-155. (10.1007/s10734-010-9329-3)

[52] Samaniego C, Lindner P, Kazmi MA, Dirr BA, Kong DT, Jeff-Eke E, Spitzmueller C, 2023 Higher research productivity=more pay? Gender pay-for-productivity inequity across disciplines. Scientometrics. **128**, 1395-1407. (10.1007/s11192-022-04513-4)

[53] Leahey E. 2007 Not by productivity alone: how visibility and specialization contribute to academic earnings. Am. Sociol. Rev. **72**, 533-561. (10.1177/000312240707200403)

[54] Wagner L, Paulitz T, Dölemeyer A, Fousse J. 2021 Jenseits der Gläsernen Decke-Professorinnen zwischen Anerkennung und Marginalisierung: Handreichung für Gleichstellungs-und Hochschulpolitik.

[55] Acker J. 1990 Hierarchies, jobs, bodies: a theory of gendered organizations. Gender Soc. **4**, 139-158. (10.1177/089124390004002002)

[56] Kozlowski D, Larivière V, Sugimoto CR, Monroe-White T. 2022 Intersectional inequalities in science. Proc. Natl Acad. Sci. USA **119**, e2113067119. (10.1073/pnas.2113067119) 34983876 PMC8764684

[57] Täuber S. 2022 Women academics' intersectional experiences of policy ineffectiveness in the European context. Frontiers in Psychology. **13**, 810569. (10.3389/fpsyg.2022.810569) 35602685 PMC9122029

[58] Barrett-Walker T, Buelow F, Te Atu O Tu MacDonald L, Brower A, James A. 2023 Stochastic modelling of intersectional pay gaps in universities. *Figshare*. 10.1098/rsos.230615 PMC10565367 37830027

